# The Influence of Different Types of Moral Stories on Honest Behaviour in Children

**DOI:** 10.3390/bs15020178

**Published:** 2025-02-08

**Authors:** Mingyue Liang, Qianqian Wang, Yanyan Zhou

**Affiliations:** 1 College of Teacher Education, Ningbo University, Ningbo 315211, China; liangmingyue@nbu.edu.cn (M.L.); 2311030007@nbu.edu.cn (Q.W.); 2 College of Education, Shandong Women’s University, Jinan 250062, China

**Keywords:** moral stories, honest behaviours, consequences of an honest story, field experiment, children

## Abstract

This study aimed to explore the influence of different types of moral stories on the honest behaviour of children aged 7–11 using a field experiment. The research findings are as follows: 1. Compared to the control condition, moral stories with positive consequences of honesty significantly promote honest behaviour in children and suppress their tendency to lie. 2. The impact of different types of moral stories on honest behaviour in children varies with age. Specifically, compared to the control condition, moral stories with positive consequences of honesty significantly promote the honest behaviour of children aged 7–10. 3. Moral stories with positive consequences of honesty significantly promote honest behaviour in children because they convey positive and encouraging messages to children that “speaking the truth leads to positive consequences”. This study suggests that teachers should utilize moral stories with positive consequences of honesty more frequently to enhance students’ honest behaviour, particularly for 7–10-year-old children.

## 1. Introduction

Honest behaviour refers to individuals displaying actions consistent with their inner thoughts, conveying truthful information to others without distorting or fabricating facts, emphasizing consistency between words and deeds, and consistency in appearance (J. X. Wu & Huang, 2012). Honesty is considered a virtue (Levine & Munguia Gomez, 2021) and an important social value (Bok, 1978) with positive regulatory effects on nonverbal behaviour in social interactions (Brambilla et al., 2016). Relevant studies indicate that children’s moral cognition regarding honesty is limited by their cognitive development, and dishonest behaviour is relatively common among elementary school students (Ran, 2005). Therefore, cultivating honest behaviour in individuals is particularly important.

The first is individual factors, including age (Talwar et al., 2016), gender (Chang, 2015), neural organization of the brain system (Yin et al., 2021), self-control and self-beliefs (Błachnio, 2021), life satisfaction (F. F. Zhang & Sun, 2022), and role expectations (W. L. Fu & Liu, 2016), etc. In response to children’s individual factors, schools can incorporate musical activities (A. Lee, 2016) and physical activities (Kim & Moon, 2004) into the curriculum, which can be combined with character education (Han, 2021) to increase children’s interest in honesty.

Second, task factors, including monetary rewards (G. Y. Fu & Wang, 2007), location of signatures, different forms of promises (H. Li et al., 2014), different verbal appeals (Talwar et al., 2015), moral stories (Talwar et al., 2016), and social norms (Liu et al., 2022). In response to these factors, scholars have researched numerous improvements, such as teachers disciplining children’s dishonest behaviours and motivating honest behaviours through creative practises, such as face-to-face teacher–student conversations (Van Zant & Kray, 2014), the use of structured conversational journals (Chan & Aubrey, 2021), questioning strategies (Wong, 2021), and telling moral stories (Biskin & Hoskisson, 1977), getting students to make promises (Kanngiesser et al., 2021), punishment reward mechanisms (Talwar et al., 2015), or implementing relevant honesty education programmes such as character education programmes (Choi & Chae, 2018), character education programme (Meewha et al., 2020), and honesty intervention programmes (Betawi, 2023), etc., to provide relevant courses and increase the number of activities to enhance the level of honesty of primary school students.

Third, environmental factors, including the gaze status of others (Zhou et al., 2018), peer observation (Isler & Gächter, 2022; Ma et al., 2018), (dis)honest behaviours of others and their consequences (Yang & Sung, 1994), different levels of social approbation (L. Y. Shi et al., 2010), different types of educational contexts (L. Y. Shi et al., 2010), educational climate and educational methods (Y. J. Li, 2017), and intimate relationships (Moon & Chang, 2010). For example, teachers can use seemingly insignificant items in the environment to prompt children to engage in honest behaviours, such as changing the position of obstacles in the environment (Zhao et al., 2020), and utilizing peer influence (Ma et al., 2018).

Among the aforementioned intervention measures, moral stories are often used as teaching tools for moral education in children (Walker & Lombrozo, 2017). Moral stories facilitate moral reflection (Sandvik, 2019) and help cultivate core moral values in preschool children (including honesty, empathy, courage, and respect) (Betawi, 2023). Storytelling as a method for conveying educational messages (Hodge et al., 2002) can promote children’s sensitivity to multiculturalism and can effectively promote sharing behaviours (Yao & Enright, 2020), helping behaviours (Du & Hao, 2018), and honest behaviours (Butean et al., 2021) among preschool children. One of the most effective ways to teach children honesty is by using morality tales. According to social learning theory, children extract more information about the value of a behaviour from the context and outcome of the observed behaviour. If the outcome of the behaviour is valuable, children are more likely to imitate the behaviour (Bandura, 1977). Therefore, when moral stories tell the positive consequences of honesty, children will be more honest (Talwar et al., 2016).

Research on moral storytelling primarily encompasses character and story types. Initially, studies on character types in moral storytelling have revealed that realistic stories are more effective than anthropomorphized stories in promoting young children’s prosocial behaviour (Larsen et al., 2018; Ding et al., 2023). In addition, research on story types in moral storytelling has revealed that moral stories with honest positive consequences effectively promote honesty in preschool children (Lee et al., 2014; Talwar et al., 2018). In other words, compared to negative and neutral story conditions, children in the positive story condition are more likely to tell the truth (Talwar et al., 2016). However, some studies suggest that emphasizing both the positive consequences of truth-telling and the negative consequences of lying can promote honest behaviour in children (Engarhos et al., 2020). In contrast, emphasizing the positive or negative consequences of admitting mistakes in moral storytelling does not significantly change children’s deceptive behaviour (Butean et al., 2021). Thus, scholars have reached different conclusions regarding the impact of different types of moral stories on honest behaviour in children (aged 3–7). The development of honest behaviour in children aged 7–11 years is of equal concern. It has been shown that dishonest behaviour occurs in children aged 3–11 years (Talwar et al., 2004) and that the tendency to lie continues to increase with age (Wilson et al., 2003) and the ability to lie increases significantly (Babu et al., 2023). Lying behaviour is common among 7–11-year-old children (K. Lee, 2013), who are able to identify lies very accurately (Peng et al., 2021). Seven–eleven-year-old Chinese children are increasingly inclined to lie (G. Y. Fu & Wang, 2007). These studies suggest that compared to 3–7-year-old children, 7–11-year-old children exhibit more dishonest behaviour, and intervening in them holds greater significance.

Piaget and Kohlberg’s theory of moral cognitive development suggests that children’s moral reasoning ability develops through a culturally universal sequence of stages (Biskin & Hoskisson, 1977), with 7–8 year-old children in the Othering and Pre-Customary stages of moral development (Change, 2012) when children are in the “punish and obey” orientation stage, making decisions to avoid punishment, and unconditionally obey authority on their own terms, rather than considering their own level. The moral development of 9–10-year-old children is in the transition from other-discipline to self-discipline (Pang & Lu, 2015) and from pre-customary to customary levels. In this period, children are in the “good boy” orientation stage, and they believe that good behaviour means helping others, making others happy, and being praised by others. Piaget noted that the age of 10 is a turning point in children’s understanding of lying, with 7–10-year-old children or younger defining lying in terms of the behaviour itself, and children 10 years old and older focusing more on the intent behind the behaviour. The motivation for pro-social lying also changes with age from self-interest to altruism (M. Q. Wu et al., 2022). Eleven-year-olds are at a stage of moral development where they are at the self-regulatory and customary levels of morality, where they are no longer based on “obedience,” and where their view of punishment changes, with no need for extra coercion to break the rules, and where they begin to give up punishment for offences in favour of rewards, and to recognize the content of wrongdoing and the nature of the punishment as consistent with it. The content of the offence is recognized as consistent with the nature of the punishment (L. D. Zhang & Wang, 2022). The developmental change in children’s understanding of moral principles between the ages of 7 and 11 is a phenomenon that deserves attention. Therefore, children aged 7–11 years were selected for the study (Evans & Lee, 2014), which is in the compulsory education stage, an important period for developing honest behaviour.

In order to easily and naturally observe whether children’s behaviour is honest or dishonest, researchers have to create conditions. The resistance to temptation paradigm pioneered by Sears et al. has been widely used in research in this field (Sears et al., 1965; Talwar & Lee, 2002; Butean et al., 2021). The main methodology of the paradigm is as follows: researchers ask children not to peek at a covered toy when they are alone in a room. Most children would break the rule because they could not contain their curiosity. When the researchers returned, they asked the children if they have peeked at the toy. The children were unaware that the hidden cameras had recorded their behaviour and were free to tell the truth or lie about their infractions (Butean et al., 2021).

Most existing research methods rely on experimental approaches, with only a few studies conducted in natural settings or using case studies or action research methods (M. Q. Wu et al., 2022). Thus, the current study employed a field experiment (Ding et al., 2019; Ding et al., 2023) using a real educational context to observe the influence of different types of moral stories (positive consequences of honesty and negative consequences of lying) on the honest behaviour of children aged 7–11. It is an important developmental stage for children’s moral principles, and their understanding of moral principles undergoes developmental changes. Previous research has revealed that moral stories with honest positive consequences effectively promote honesty in preschool children (Lee et al., 2014; Talwar et al., 2018). Therefore, this study hypothesized that moral stories with honest positive consequences would promote honest behaviours in 7–11-year-old children.

For ease of reading, the main elements of the two studies are presented here first. Experiment 1 conducted a one-way, 3-level, between-subjects experimental design to explore differences in honest behaviour in children between morality tales that emphasized the positive consequences of honesty, morality tales that emphasized the negative consequences of lying, and a neutral condition, and found that morality tales with positive consequences of honesty had a facilitating effect on the honest behaviour of 7–10 year-olds, and an inhibiting effect on their lying behaviour. To investigate whether it was the stories themselves that made children behave honestly or whether the stories emphasized the positive consequences of honesty, this study conducted a second experiment. It was found that moral stories with positive consequences of honesty significantly promoted honest behaviour because the stories emphasized the positive consequences of honesty and conveyed the message that “when one tells the truth, there are positive consequences.”

## 2. Experiment 1

### 2.1. Experimental Purpose

Experiment 1 aimed to investigate the influence of moral stories with different behavioural consequences on the honest behaviour of 7–11-year-old children in a temptation resistance task. Two research hypotheses were proposed. Hypothesis 1: Moral stories with honest positive consequences promote honest behaviour in children. Hypothesis 2: Different types of moral stories have age-related differences regarding their influence on honest behaviour in children.

### 2.2. Participants

Based on the single-factor three-level between-subjects experimental design. As suggested by Cohen (1988), the expected alpha value was set to 0.05, the statistical validity was set to 0.80, and the effect size was set to the medium level f = 0.25. Based on these criteria, the minimum sample size required was calculated using G*Power 3.0.10 and found to be 159. To ensure that the final number of valid participants met the minimum sample size requirement, 165 7–11-year-old children were randomly selected from four primary schools in China. The schools were located in Ningbo, Zhejiang Province. Due to personal reasons such as children taking leave, leaving, etc., 5 invalid subjects were excluded and the final number of valid subjects was 160 (M = 8.59, SD = 1.42, 86 boys, 74 girls). According to the actual age distribution of the selected schools, a stratified sampling method was used, so that a different number of people were drawn out at each age stage. Among them, there were 48 participants aged 7 (M = 7.45, SD = 0.23), 43 aged 8 (M = 8.44, SD = 0.22), 23 aged 9 (M = 9.59, SD = 0.18), 23 aged 10 (M = 10.56, SD = 0.21), and 24 aged 11 (M = 11.58, SD = 0.26). This study was agreed upon by the subjects as well as the guardians of the subjects, and the parents signed an informed consent form for the purpose and content of the study. At the end of the experiment, each student was paid accordingly.

### 2.3. Experimental Materials

#### 2.3.1. Moral Stories

In this study, three stories, “The Tortoise and the Hare”, “George Washington and the Cherry Tree”, and “The Boy Who Cried Wolf”, were presented to the children in the form of PowerPoint slides. This approach was conducted to control the time spent reading the stories and to make the stories more engaging through the researcher’s narration. Each story comprised 7 PowerPoint slides and took approximately 3 min to tell.

The control group read a nonmoral story, “The Tortoise and the Hare” (control). Previous research has clearly identified “The Tortoise and the Hare” as a nonmoral story that does not contain moral information or a moral message regarding how to interact with others (Du et al., 2017). The story is unrelated to lying or telling the truth. The experimental groups read moral stories, namely, “George Washington and the Cherry Tree” (positive consequences of honesty) and “The Boy Who Cried Wolf” (negative consequences of lying). “George Washington and the Cherry Tree” emphasizes the positive consequences of honesty. When George Washington tells his father the truth about chopping down the cherry tree, his father praises his honesty. The value logic of the story is “be honest—honest people will be rewarded” (Z. Y. Shi, 2009). “The Boy Who Cried Wolf” emphasizes the negative consequences of lying. The shepherd boy frequently lies by saying that the wolf is coming, but when the wolf actually appears, nobody believes him, and his sheep are eaten by the wolf. The value logic of the story is “do not lie—lying people will be punished” (Z. Y. Shi, 2009). These three stories have been used in previous research on moral stories (Lee et al., 2014).

#### 2.3.2. Temptation Resistance Paradigm

**Figure 1:**
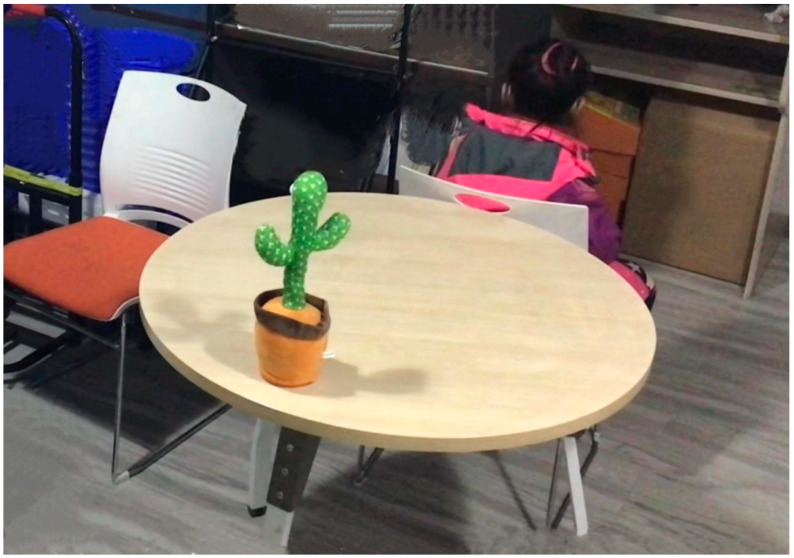
Arrangement of the experimental setting.

Previous research has shown that the temptation resistance paradigm is often used to measure children’s lying behaviour (Newton et al., 2000). Therefore, in this study, based on the temptation resistance paradigm, a game-like task was used to measure honest behaviour in children. Common animals and their sounds familiar to children were selected, including a pig toy that makes an “oink oink” sound, a cat toy that makes a “meow meow” sound, a duck toy that makes a “quack quack” sound, and a toy with various indistinguishable sounds (a “dancing cactus”) (see Figure 1).

### 2.4. Experimental Procedure

**Figure 2:**
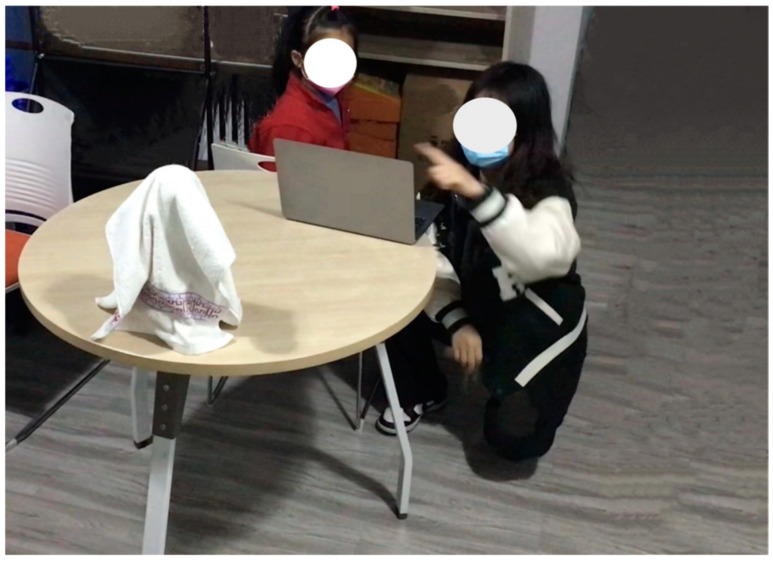
Experimental procedure.

This study employed an on-site experimental method, with the duration of each experiment controlled at approximately 10 min. Each child was observed individually. The experimenter took the child to a classroom equipped with a concealed camera and engaged in a “guessing game” with the child (see Figure 2). The child sat on a chair facing away from the table, and the experimenter placed toy figures of a pig, a duck, and a cat on the table one by one. Each toy emitted a sound, and the child had to guess the toy based on the sounds “oink oink” “quack quack” and “meow meow”. After three rounds of the game, the experimenter introduced a cactus toy (the target toy) and played various songs, such as “Happy Birthday”, “I Will Be a Great Hero”, and “Take Me To Church”, through the cactus toy. Since children rarely encounter objects that produce multiple sounds in real life, it was difficult for them to guess the answer “cactus toy” based on these sounds. While the child was guessing the answer, the experimenter made an excuse to leave the room, instructing the child not to turn around and peek. After 60 s, the experimenter returned to the room and covered the cactus toy with a white cloth. Then, the experimenter randomly selected one of the three stories (“The Tortoise and the Hare”, “George Washington and the Cherry Tree”, and “The Boy Who Cried Wolf”) and told it to the child. Afterwards, the experimenter asked the child about the key events in the story to ensure the effectiveness of the intervention.

Under control conditions, the experimenter asked the children, “I’m going to ask you a question, and I want you to tell me the truth, okay?” After obtaining the child’s agreement, the experimenter asked, “After I leave the room, did you turn around and peek at the toy?”

Under the condition of negative consequences of lying, the experimenter asked the children, “I’m going to ask you a question, and I don’t want you to be like the boy who cried wolf in the story. I want you to tell me the truth, okay?” After obtaining the child’s agreement, the experimenter asked, “After I left the room, did you turn around and peek at the toy?”

Under the condition of positive consequences of honesty, the experimenter asked the children, “I’m going to ask you a question, and I want you to tell me the truth, just like George Washington in the story, okay?” After obtaining the child’s agreement, the experimenter asked, “After I leave the room, did you turn around and peek at the toy?”

### 2.5. Data Encoding

By combining video recordings and observing whether the children’s reports matched their behaviours, the following behaviours were defined as honest behaviour: “Children who did not peek at the target toy after the experimenter left and admitted to not peeking after hearing the story” and “Children who peeked at the target toy after the experimenter left and admitted to peeking after hearing the story”. The behaviour of “Children who peeked at the target toy after the experimenter left but denied peeking after hearing the story” was defined as lying behaviour^1^.

The two indicators used to assess whether children peeked at the toy behind them are as follows: 1. After the experimenter left, the child turned their head more than 90 degrees while keeping their eyes open and looked at the toy behind them (Talwar & Lee, 2002, 2008). 2. After the experiment, the recorded video was reviewed to confirm whether the child’s gaze lingered on the target toy behind them. In order to prevent the subjective element of the experimenter’s personal judgement from adversely affecting the results of the experiment, so that we can determine whether the children’s behaviour was lying behaviour or honest behaviour, we used the Experts Grading Method. We invited two experts who were unaware of the experiment’s purpose to independently watch the recorded videos and code the children’s behaviours. Honest and lying behaviours were assigned values of 0 and 1, respectively, and then the consistency of the expert coding was tested (see Table 1).

The test results indicated 160 valid cases without missing data, resulting in a total sample size of 160. Scott’s pi coefficient was 0.988, indicating a high level of consistency. This finding suggests that the two coders had consistent opinions regarding the classification of the two behaviours for most of the children, with only a few cases showing disagreement in the classification of behaviours.

### 2.6. Data Processing and Results Analysis

SPSS 22.0 was used to process the experimental data, employing the chi-square test for independence in nonparametric analysis. Foremost, the influence of different types of moral stories on honest behaviour in children was analyzed. Then, this study examined whether there were age differences in the influence of different types of moral stories on honest behaviour in children.

#### 2.6.1. Analysis of the Impact of Different Behavioural Consequences on Moral Stories

The researchers used Pearson’s chi-squared test to analyze the association between honest behaviour and lying behaviour in the conditions of positive consequences of honesty and control conditions. Additionally, the researchers analyzed the association between lying behaviour and honest behaviour in the conditions of negative consequences of lying and control conditions.

**Figure 3:**
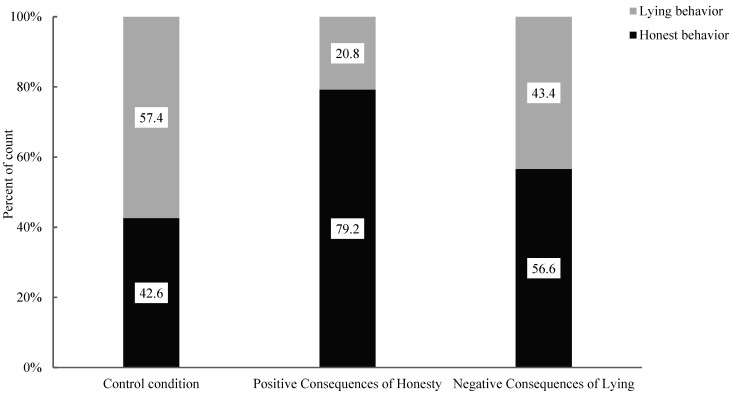
Percentages of children’s behaviour under control, positive consequences of honesty and negative consequences of lying conditions.

First and foremost, the researchers analyzed the association between honest behaviour and lying behaviour in the conditions of positive consequences of honesty and control conditions (see Figure 3). The results showed a significant correlation between the positive consequences of the honesty condition and the control condition with children’s honest and lying behaviours (χ^2^ (1, 107) = 15.07, *p* < 0.001). Specifically, there was a significant difference in the proportion of honest behaviour in children (Positive Consequences of Honesty: 79.2% vs. Control: 42.6%) and lying behaviour (Positive Consequences of Honesty: 20.8% vs. Control: 57.4%) between the two conditions. This finding indicates that, compared to the control condition, the positive consequences of the honesty condition can influence honest behaviour in children. Post hoc testing was further conducted to analyze this effect, as recommended by Agresti (2012). When the absolute value of the adjusted standardized residual exceeds 2, the difference between the observed frequency and the expected frequency can be considered significant. The analysis (see Table 2) showed that the absolute value of the adjusted standardized residual for honest behaviour was 3.9 and that for lying behaviour was 3.9, both exceeding 2. This finding indicates that moral stories with positive consequences of honesty can significantly enhance honest behaviour in children and suppress their lying behaviour.

Furthermore, the researchers analyzed the association between honest behaviour and lying behaviour in the conditions of negative consequences of lying and control conditions (see Figure 3). The results revealed no significant correlation between the negative consequences of lying condition and the control condition with children’s honest and lying behaviours (χ^2^ (1, 107) = 2.10, *p* = 0.147). Specifically, there was no significant difference in the proportion of honest behaviour in children (Negative Consequences of Lying: 46.9% vs. Control: 28.1%) and lying behaviour (Negative Consequences of Lying: 53.1% vs. Control: 71.9%) between the two conditions. Thus, compared to the control condition, moral stories with negative consequences of lying do not affect children’s honest and lying behaviours.

The above results indicate that different types of moral stories have different effects on honest behaviour in children. Specifically, moral stories with positive consequences of honesty significantly enhance honest behaviour in children and suppress their lying behaviour, while moral stories with negative consequences of lying do not affect children’s honest or lying behaviours.

#### 2.6.2. Analysis of the Effects of Moral Stories with Different Consequences on Children’s Behaviour Across Different Age Groups

**Figure 4:**
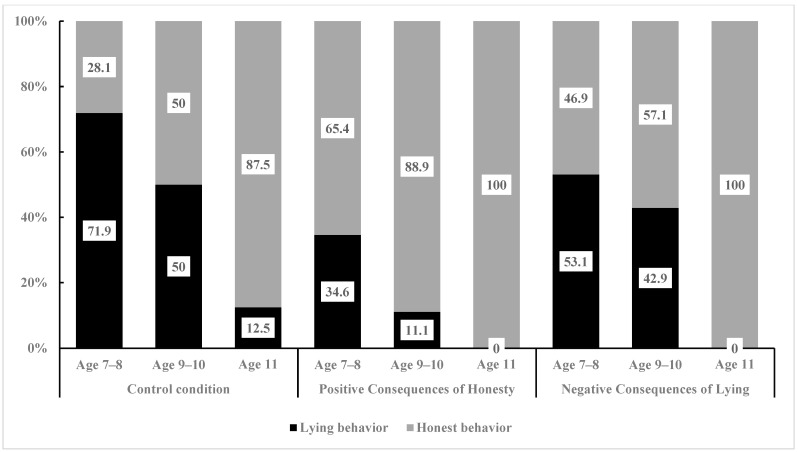
Percentage of children’s behaviour in the control condition, honesty positive consequences condition, and lying negative consequences condition at different ages.

Due to the finding that moral stories with positive consequences of honesty significantly enhance honest behaviour in children and suppress their lying behaviour compared to the control condition, the researchers further investigated whether different types of moral stories are associated with age differences in their effects on honest behaviour in children. Therefore, we divided the primary school age into three stages. Then, we used Fisher’s exact test to compare the differences in the number of children displaying honest behaviour and lying behaviour under control, positive consequences of honesty, and negative consequences of lying conditions among 7- to 8-year-old children, 9- to 10-year-old children, and 11-year-old children (see Figure 4).

To begin with, the researchers analyzed the effects of moral stories with different consequences on honest behaviour among 7- to 8-year-old children. They examined the association between the positive consequences of the honesty condition and the control condition with honest and lying behaviours (see Figure 4). A significant difference in the proportion of children’s honest and lying behaviours was found (χ^2^ (1, 58) = 8.05, *p* = 0.008). Specifically, there was a significant difference in the proportion of honest behaviour in children (Positive Consequences of Honesty: 65.4% vs. Control: 28.1%) and lying behaviour (Positive Consequences of Honesty: 34.6% vs. Control: 71.9%). Compared to the control condition, moral stories with positive consequences of honesty significantly affected the honest behaviour and lying behaviour of 7- to 8-year-old children. Post hoc testing was conducted to further analyze this effect, and the results (see Table 3) showed that the absolute value of the adjusted standardized residual for honest behaviour was 2.8 and that for lying behaviour was 2.8, both exceeding 2. Thus, moral stories with positive consequences of honesty can significantly enhance honest behaviour and suppress lying behaviour among 7- to 8-year-old children.

**Figure 5:**
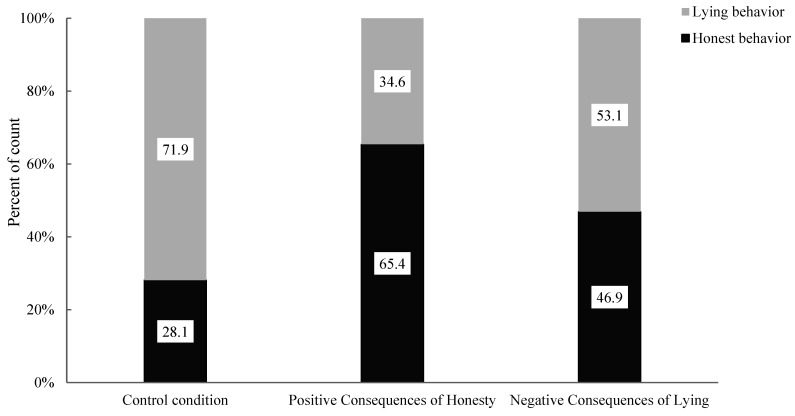
Percentages of behaviours in 7–8-year-old children under the control condition, positive consequences of honesty condition and negative consequences of lying condition.

The researchers then analyzed the association between honest behaviour and lying behaviour in 7- to 8-year-old children under the negative consequences of lying condition and the control condition (see Figure 5). The results indicated no significant difference in the proportions of honest and lying behaviours in 7- to 8-year-old children (χ^2^ (1, 64) = 2.40, *p* = 0.196). Specifically, there was no significant difference in the proportions of honest behaviour (Negative Consequences of Lying: 62.5% vs. Control: 37.5%) and lying behaviour (Negative Consequences of Lying: 42.5% vs. Control: 57.5%) between the two conditions. Compared to the control condition, the negative consequences of lying moral stories did not significantly influence honest and lying behaviours among 7- to 8-year-old children.

**Figure 6:**
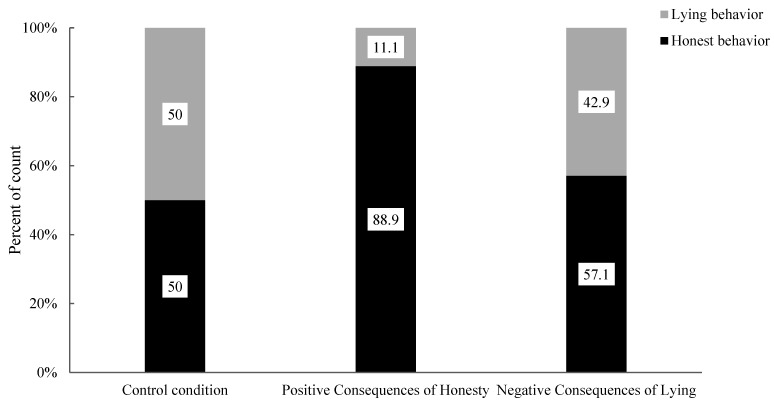
Percentages of behaviour in 9- to 10-year-old children under control, positive consequences of honesty and negative consequences of lying conditions.

Next, the researchers analyzed the impact of moral stories with different behaviour consequences on the honest behaviour of 9- to 10-year-old children. The association between the positive consequences of the honesty condition and the control condition regarding honest and lying behaviours was examined (see Figure 6). The results revealed a significant difference in the proportions of honest and lying behaviours in 9- to 10-year-old children (χ^2^ (1, 32) = 5.89, *p* = 0.022). Specifically, there was a significant difference in the proportions of honest behaviour (Positive Consequences of Honesty: 88.9% vs. Control: 50.0%) and lying behaviour (Positive Consequences of Honesty: 11.1% vs. Control: 50.0%) between the two conditions. Compared to the control condition, moral stories with positive consequences of honesty significantly impacted the honest behaviour and lying behaviour among 9- to 10-year-old children. Post hoc testing was further used to analyze this effect, and the results (see Table 4) indicated that the adjusted standardized residuals for honest behaviour and lying behaviour, both exceeded 2 (2.4 for both). Thus, moral stories with positive consequences of honesty significantly enhanced honest behaviour and inhibited lying behaviour in 9- to 10-year-old children.

The researchers then analyzed the association between honest behaviour and lying behaviour in 9- to 10-year-old children under the negative consequences of lying condition and the control condition (see Figure 5). The results revealed no significant difference in the proportions of honest and lying behaviours in 9- to 10-year-old children (χ^2^ (1, 28) = 0.14, *p* = 1.00). Specifically, there was no significant difference in the proportions of honest behaviour (Negative Consequences of Lying: 57.1% vs. Control: 50.0%) and lying behaviour (Negative Consequences of Lying: 42.9% vs. Control: 50.0%) between the two conditions. Compared to the control condition, moral stories with negative consequences of lying did not have a significant impact on honest and lying behaviours among 9- to 10-year-old children.

**Figure 7:**
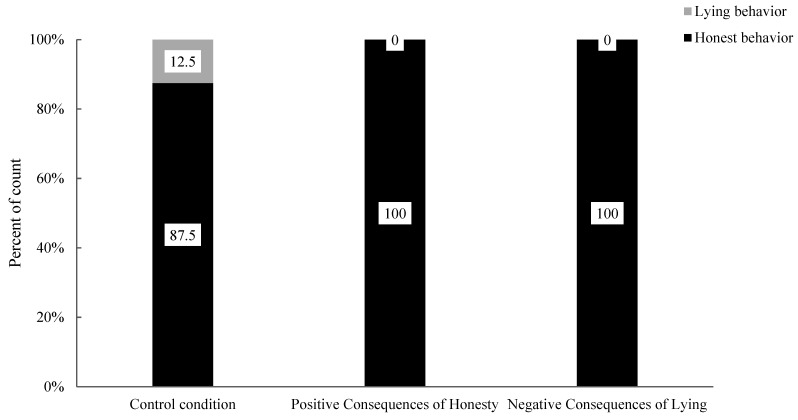
Percentages of behaviour in 11-year-old children under control, positive consequences of honesty and negative consequences of lying conditions.

Finally, the researchers analyzed the impact of moral stories with different behaviour consequences on honest behaviour in 11-year-old children. The association between honest and lying behaviours in 11-year-old children was assessed under the positive consequences of the honesty condition and the control condition (see Figure 7). The analysis results showed that there was no significant difference in the proportions of honest behaviour and lying behaviour in 11-year-old children (χ^2^ (1, 17) = 1.20, *p* = 0.471). Specifically, there was no significant difference in the proportions of honest behaviour (positive consequences of honesty: 100.0% vs. control: 87.5%) and lying behaviour (positive consequences of honesty: 0.0% vs. control: 12.5%) between the two conditions. Compared to the control condition, moral stories with positive consequences of honesty did not significantly impact the honest behaviour of 11-year-old children. The differences in honest and lying behaviours in 11-year-old children were also assessed between the negative consequences of lying condition and the control condition (see Figure 6). The analysis revealed no significant difference in the proportions of honest behaviour and lying behaviour among 11-year-old children (χ^2^ (1, 15) = 0.94, *p* = 1.00). Specifically, there was no significant difference in the proportions of honest behaviour (Negative Consequences of Lying: 100.0% vs. Control: 87.5%) and lying behaviour (Negative Consequences of Lying: 0.0% vs. Control: 12.5%) between the two conditions. Compared to the control condition, moral stories with negative consequences of lying did not significantly impact the honest behaviour of 11-year-old children.

The above results indicate that the influence of different types of moral stories on honest behaviour in children varies with age. Specifically, compared to the control condition, there were significant differences in the proportions of honest behaviour and lying behaviour in 7- to 10-year-old children under the positive consequences of honesty condition. However, there were no significant differences in the proportions of honest and lying behaviours in 11-year-old children. Furthermore, compared to the control condition, there were no significant differences in the proportions of honest and lying behaviours in 7- to 11-year-old children under the negative consequences of lying condition. In other words, relative to the control condition, moral stories with positive consequences of honesty had a promoting effect on the honest behaviour of 7- to 10-year-old children and an inhibitory effect on their lying behaviour. Moral stories with negative consequences of lying did not significantly impact the honest behaviour of 7- to 10-year-old children. Additionally, relative to the control condition, moral stories with positive consequences of honesty and moral stories with negative consequences of lying did not significantly impact the honest behaviour of 11-year-old children.

## 3. Experiment 2

### 3.1. Objective

Experiment 1 revealed that moral stories emphasizing the positive consequences of honesty significantly increased honest behaviour and suppressed lying behaviour among children aged 7–11 compared to the control condition. Thus, it was necessary to verify that the reason moral stories promote honest behaviour in children is the emphasis on the positive consequences of honesty rather than the story itself. In other words, compared to “The Boy Who Cried Wolf” and “The Tortoise and the Hare”, the story “George Washington and the Cherry Tree” promotes honest behaviour in children because it emphasizes the positive consequences of honesty. Therefore, in Experiment 2, “George Washington and the Cherry Tree” was adapted by changing the ending to reflect the negative consequences of lying. Specifically, after George Washington chopped down his father’s cherry tree without telling the truth, his father was disappointed when he discovered the truth and confiscated George’s axe. All other content remained unchanged. The hypothesis of the experiment was that when morality tales emphasize the negative consequences of lying, they will fail to promote honest behaviour in children.

### 3.2. Participants

A within-subjects experimental design with a single factor and three levels was used in this study. Fifty participants aged 7–11 years (M = 8.84, SD = 1.64) were randomly selected from four primary schools in Ningbo, Zhejiang Province, China.

### 3.3. Experimental Materials

The adapted honesty-associated story “George Washington and the Cherry Tree” was used in this experiment.

### 3.4. Experimental Procedure

The procedures and materials of Experiment 1 and Experiment 2 were similar, except for the moral story used in the negative consequences of lying condition. In Experiment 1, the moral story used was “The Boy Who Cried Wolf”, while in Experiment 2, the moral story used was the adapted honesty-associated story “George Washington and the Cherry Tree”.

### 3.5. Data Coding

Since the moral story with positive consequences of honesty significantly promoted honest behaviour in children in Experiment 1, it is only necessary to compare the honest behaviour of the experimental group under the negative consequences of lying condition in Experiment 2 with the control group. The data coding requirements remained consistent with Experiment 1. Two experts who were unaware of the experimental objectives were invited to watch the recorded experiments and code the children’s behaviour. Honest and lying behaviours were assigned values of 0 and 1, respectively. The consistency of coding between the two experts was then tested (see Table 5).

The test results indicated 104 valid data points without missing data, with a total sample size of 104. Scott’s pi coefficient was 0.981, indicating a high level of consistency. This finding suggests that the two coders had consistent opinions in categorizing the behaviours of most children, with only a few disagreements in classifying the behaviours of a small number of children.

### 3.6. Data Processing and Results Analysis

#### 3.6.1. Analysis of the Effects of Different Behavioural Consequences in the Same Moral Story

**Figure 8:**
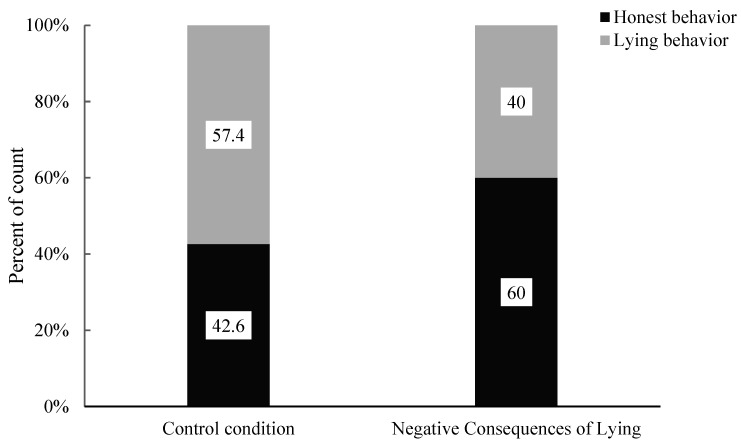
Percentage of children’s behaviour under the control condition and negative consequences of lying condition.

The researchers conducted Pearson’s chi-squared test to analyze the association between the consequences of negative lying and the control conditions on honest behaviour in children and lying behaviour (see Figure 8). The results revealed no significant correlation between the two conditions and children’s honest and lying behaviours (χ^2^ (1, 104) = 3.15, *p* = 0.076). Specifically, there were no significant differences in the proportion of honest behaviour in children (Negative Consequences of Lying: 60% vs. Control: 42.6%) and lying behaviour (Negative Consequences of Lying: 40% vs. Control: 57.4%). The moral story emphasizing the negative consequences of lying did not significantly impact honest behaviour in children and lying behaviour compared to the control condition.

#### 3.6.2. Analysis of the Effects of Different Behavioural Consequences in the Same Moral Story on Children of Different Age Groups

**Figure 9:**
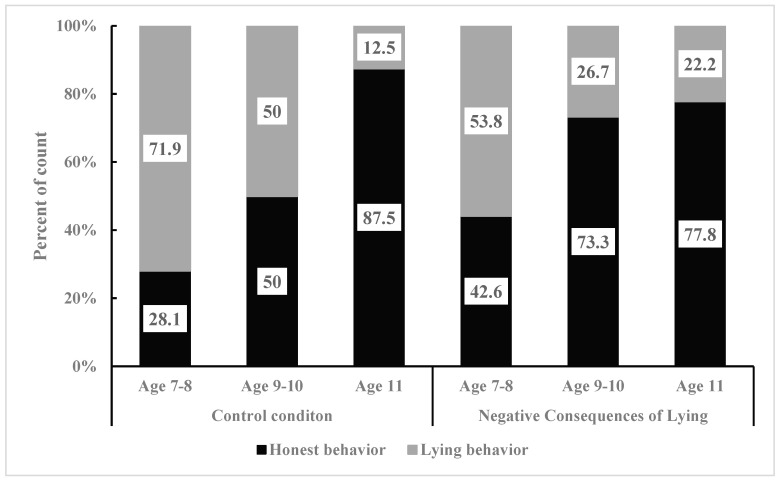
Percentage of children’s behaviour in the control condition and the lying negative consequences condition, by age group.

To compare the effectiveness of different morality tales on children’s behaviour at different ages, this study compared the number of children within different age groups in terms of their performance of honest behaviour and lying behaviour (see Figure 9).

**Figure 10:**
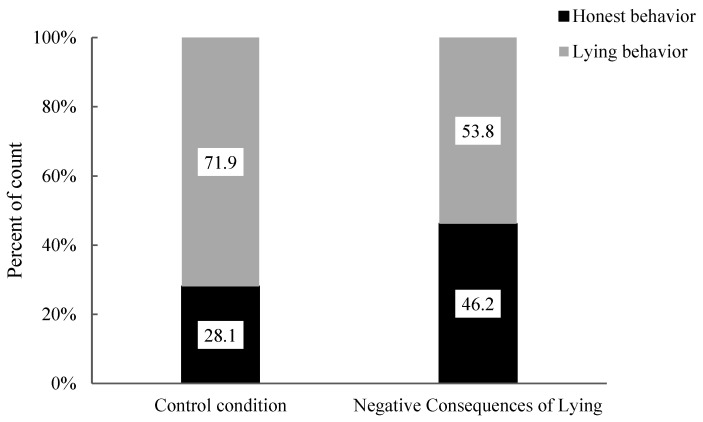
Percentage of 7–8-year-old children’s behaviour in the control condition and the lying negative consequences condition.

At the outset, the researchers used Fisher’s exact test to analyze the association between the consequences of negative lying and the control conditions on the honest behaviour and lying behaviour of 7- to 8-year-old children (see Figure 10). The results revealed no significant difference in the proportion of honest behaviour and lying behaviour among 7- to 8-year-old children (χ^2^ (1, 58) = 2.02, *p* = 0.18). Specifically, there were no significant differences in the proportion of honest behaviour (Negative Consequences of Lying: 42.6% vs. Control: 28.1%) and lying behaviour (Negative Consequences of Lying: 53.8% vs. Control: 71.9%) among the children. The moral story emphasizing the negative consequences of lying did not significantly impact honest behaviour and lying behaviour among 7- to 8-year-old children compared to the control condition.

**Figure 11:**
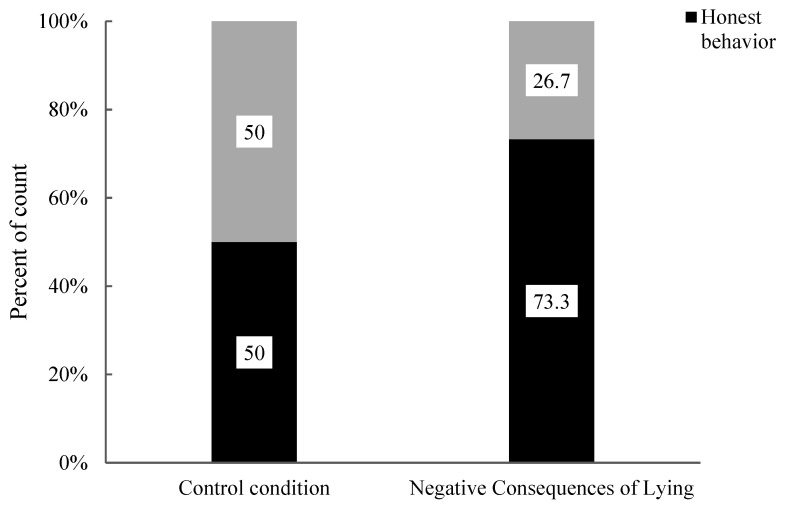
Percentage of behaviours of 9- to 10-year-old children under the control condition and negative consequences of lying condition.

Then, the researchers used Fisher’s exact test to analyze the association between the consequences of negative lying and the control conditions on the honest behaviour and lying behaviour of 9- to 10-year-old children (see Figure 11). The results revealed no significant difference in the proportion of honest behaviour and lying behaviour among 9- to 10-year-old children (χ^2^ (1, 29) = 1.68, *p* = 0.264). Specifically, there were no significant differences in the proportion of honest behaviour (Negative Consequences of Lying: 73.3% vs. Control: 50.0%) and lying behaviour (Negative Consequences of Lying: 26.7% vs. Control: 50.0%) among the children. The moral story emphasizing the negative consequences of lying did not significantly impact honest behaviour and lying behaviour among 9- to 10-year-old children compared to the control condition.

**Figure 12:**
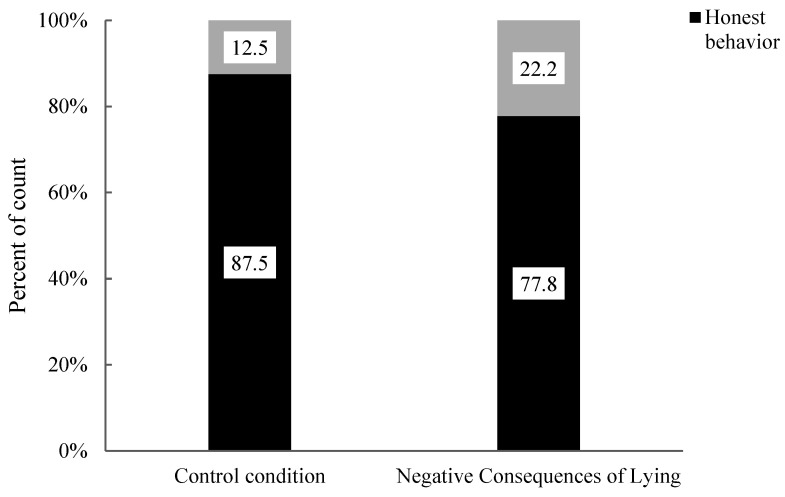
Percentage of behaviours of 11-year-old children under control condition and negative consequences of lying condition.

Finally, Fisher’s exact test was employed to analyze the association between the honest behaviour and lying behaviour of 11-year-old children under the negative consequences of lying condition and the control condition (see Figure 12). The analysis revealed no significant difference in the proportion of 11-year-old children’s honest and lying behaviours (χ^2^ (1, 17) = 0.28, *p* = 1.00). Specifically, there was no significant difference in the proportion of honest behaviour in children (Negative Consequences of Lying: 77.8% vs. Control: 87.5%) and lying behaviour (Negative Consequences of Lying: 22.2% vs. Control: 12.5%). The moral story emphasizing the negative consequences of lying did not significantly impact honest behaviour and lying behaviour among 11-year-old children compared to the control condition.

The above results indicate that compared to the control condition, moral stories emphasizing the positive consequences of honesty significantly promote honest behaviour among children aged 7–11. In contrast, they have an inhibitory effect on their lying behaviour. The impact of different types of moral stories on honest behaviour in children varies with age. Specifically, compared to the control condition, moral stories emphasizing the positive consequences of honesty have a promoting effect on the honest behaviour of children aged 7–10 and an inhibitory effect on their lying behaviour; however, they do not significantly affect the honest behaviour of 11-year-old children. Compared to the control condition, moral stories emphasizing the negative consequences of lying do not significantly impact the honest behaviour of 7–11-year-old children. Furthermore, the significant promotion of honest behaviour in children by moral stories emphasizing the positive consequences of honesty is attributed to stories conveying the message that telling the truth leads to positive consequences.

## 4. Discussion

This study utilized a field experiment to examine the effects of different types of moral stories on the honest behaviour of 7–11-year-old children. The results showed the following: 1. Moral stories emphasizing the positive consequences of honesty significantly promoted honest behaviour in children, while moral stories emphasizing the negative consequences of lying did not significantly affect their honest behaviour. The impact of different types of moral stories on honest behaviour in children varied with age. Specifically, moral stories emphasizing the positive consequences of honesty had a significant promoting effect on the honest behaviour of 7–11-year-old children but did not significantly affect the honest behaviour of 11-year-old children. Moral stories emphasizing the negative consequences of lying did not significantly impact the honest behaviour of 7–11-year-old children. 3. The key narrative element that facilitated the promotion of honest behaviour in children in moral stories was emphasizing the positive consequences of honest actions. This finding is consistent with previous research.

Furthermore, the influence of different types of moral stories on honest behaviour in children varied with age. Specifically, compared to the control condition, moral stories emphasizing the positive consequences of honesty had a significant promoting effect on the honest behaviour of 7–10-year-old children but did not significantly affect the honest behaviour of 11-year-old children. Moral stories emphasizing the negative consequences of lying did not significantly impact the honest behaviour of children aged 7–11. According to Piaget’s theory of moral cognitive development and Kohlberg’s cognitive theory of moral development, 7–8 year-old children are in the orientation stage of “punishment and obedience”, they are in favour of the punishment of fait accompli, they believe that the act of being punished is bad in itself, and they are easily affected by the consequences of the characters’ behaviour in the morality stories; therefore, the morality stories with negative consequences of lying will not significantly increase their honest behaviour, while the morality stories with positive consequences of honesty will significantly increase their honest behaviour. Therefore, moral stories with negative consequences of lying will not significantly increase their honest behaviour, while moral stories with positive consequences of honesty will significantly increase their honest behaviour. Nine- to ten-year-old children are in the orientation stage of “good boy”, they believe that a good behaviour is one that helps others, makes others happy, and is praised by others. Children at the age of 11 have changed their view of punishment and do not need to be coerced to break the rules, so neither moral stories with positive consequences for honesty nor those with negative consequences for lying have a significant effect on their honest behaviour.

Regarding previous research, this study revealed that moral stories emphasizing the positive consequences of honesty significantly promote the honest behaviour of elementary school children, thus extending previous research on preschool children aged 3–7 (Lee et al., 2014). However, this study has certain limitations. First of all, it only used “Washington and the Cherry Tree” as a moral story emphasizing the positive consequences of honesty, with verbal rewards as the positive consequence. Further exploration is warranted to examine the effects of different degrees of positive consequences, such as material rewards or verbal rewards, on honest behaviour in children. In addition, although this study identified age differences in the impact of different types of moral stories on honest behaviour in children, it did not deeply analyze the reasons behind these differences. Future research could analyze the causes of these differences from psychological and neuroscientific perspectives. Furthermore, previous research indicates that parenting styles and marital status can influence honest behaviour in children (Malloy et al., 2019; Tobol & Yaniv, 2019; Dykstra et al., 2020). Some children may have received prior education on honesty from their parents, which could have affected their behaviour during the experimental process. However, this study did not consider the influence of parents on honest behaviour in children and did not collect information about the participating children’s family backgrounds. Future research could incorporate this factor as an influencing variable. Lastly, the sample size of our second study was insufficient, which may have affected the generalizability of the findings. Therefore, we will continue to expand the sample size to verify the veracity of the findings in the future.

## 5. Conclusions

The results revealed the following: 1. Moral stories that emphasized the positive outcomes of honesty had a significant impact on promoting honest behaviour in children, while moral stories emphasizing the negative outcomes of lying did not have a significant effect. The impact of different types of moral stories on honest behaviour in children varied with age. Specifically, moral stories emphasizing the positive consequences of honesty had a significant promoting effect on the honest behaviour of 7–10-year-old children, but did not on 11-year-old children. Moral stories emphasizing the negative consequences of lying did not significantly impact the honest behaviour of 7–11-year-old children. Emphasizing the positive consequences of honest behaviour is a key narrative element in moral stories that promotes honest behaviour in children. The key narrative element that facilitated the promotion of honest behaviour in children in moral stories was emphasizing the positive consequences of honest actions.

## Figures and Tables

**Table 1:** Consistency test of data encoding.

Demonstration Conditions		Frequency (Percentage)	
		Honest Behaviour	Lying Behaviour
Control (n = 54)	Rater 1	23 (42.59%)	31 (57.41%)
	Rater 2	23 (42.59%)	31 (57.41%)
Positive Consequences of Honesty (n = 53)	Rater 1	42 (79.25%)	11 (20.75%)
	Rater 2	41 (77.36%)	12 (22.64%)
Negative Consequences of Lying (n = 53)	Rater 1	30 (56.60%)	23 (43.40%)
	Rater 2	29 (54.72%)	24 (45.28%)

**Table 2:** Cross-analysis of the effects of positive consequences of honesty and control conditions on children’s behaviour.

Condition	Children’s Behaviour	
	Honest	Lying
Control	23 (−3.9)	31 (3.9)
Positive Consequences of Honesty	41 (3.9)	11 (−3.9)

Note. Adjusted residuals appear in parentheses below the observed frequencies.

**Table 3:** Cross-analysis of the effects of positive consequences of honesty and control conditions on the behaviour of 7- to 8-year-old children.

Condition	Children’s Behaviour (Age = 7–8)	
	Honest	Lying
Control	9 (−2.8)	23(2.8)
Positive Consequences of Honesty	17 (2.8)	9(−2.8)

**Table 4:** Cross-analysis of the impact of the positive consequences of the honesty condition and the control condition on children’s behaviour.

Condition	Children’s Behaviour (Age = 9–10)	
	Honest	Lying
Positive Consequences of Honesty	16 (−2.4)	2 (2.4)
Control	7 (2.4)	7 (−2.4)

**Table 5:** Consistency test of data coding.

Demonstration Conditions		Frequency (Percentage)	
		Honest Behaviour	Lying Behaviour
Control (n = 54)	Rater 1	23 (42.59%)	31 (57.41%)
	Rater 2	23 (42.59%)	31 (57.41%)
Negative Consequences of Lying (n = 50)	Rater 1	30 (56.60%)	20 (43.40%)
	Rater 2	29 (54.72%)	21 (45.28%)

## Data Availability

We have shared the research data on Mendeley Data, and the data link is as follows: https://data.mendeley.com/drafts/6xfs9hdnfk. Accessed on 22 November 2024.

## Note

1 The children in the experiment did not exhibit the behaviour of “Children who did not peek at the target toy after the experimenter left but denied peeking after hearing the story” thus, lying behaviour in this study included only one behaviour.
